# Impact of Diabetes Mellitus on Human Erythrocytes: Atomic Force Microscopy and Spectral Investigations

**DOI:** 10.3390/ijerph15112368

**Published:** 2018-10-26

**Authors:** Mohamad S. AlSalhi, Sandhanasamy Devanesan, Khalid E. AlZahrani, Mashael AlShebly, Fatima Al-Qahtani, Karim Farhat, Vadivel Masilamani

**Affiliations:** 1 Department of Physics and Astronomy, College of Science, King Saud University, Riyadh 11451, Saudi Arabia; malsalhy@gmail.com (M.S.A.); alzkhalid@ksu.edu.sa (K.E.A.); 2 Research Chair in Laser Diagnosis of Cancers, Department of Physics and Astronomy, College of Science, King Saud University, Riyadh 11451, Saudi Arabia; 3 King Abdullah Institute for Nanotechnology, King Saud University, Riyadh 1451, Saudi Arabia; 4 Department of Obstetrics and Gynecology, College of Medicine, King Khalid University Hospital, King Saud University, Riyadh 11451, Saudi Arabia; shebaili@yahoo.com; 5 Hematology Unit, Department of Pathology, College of Medicine, King Saud University and King Saud University Medical City, Riyadh 11451, Saudi Arabia; fatma.qahtani@yahoo.com; 6 Cancer Research Chair, College of Medicine, King Saud University, Riyadh 11451, Saudi Arabia; farhatscience@yahoo.fr

**Keywords:** Eythrocytes morphology, atomic force microscopy (AFM), spectral analysis, fluorescent bio-molecules, diabetes-induced cardiovascular diseases

## Abstract

Diabetes mellitus (DM) is a common metabolic disease indicated by high sugar levels in the blood over a prolonged period. When left untreated, it can lead to long-term complications, such as cardiovascular disease, stroke, and diabetic retinopathy or foot ulcers. Approximately 415 million people (about 8.3% of the world’s population) had diabetes worldwide in 2015, with 90% of the cases classified as Type 2 DM, which is caused by insulin resistance that arises mostly from being overweight and from a lack of exercise. DM affects every part of the body, including the erythrocytes. The aim of the present report is to gain insight into the damage done to the erythrocytes of patients classified with pre-diabetes and diabetes (plenty are found in the Kingdom of Saudi Arabia, a country where young people encompass a large segment of the population). The study presents results on the morphological analysis of erythrocytes by atomic force microscopy (AFM) and molecular investigations by fluorescence spectroscopy (FS). Our results indicate significant differences (in the morphology, size, and hemolytic end products) between the erythrocytes of diabetic patients (HbA1C, glycated hemoglobin, levels of 8–10%) and normal controls. It is well-known that DM and smoking are two major contributory factors for cardiovascular diseases (CVDs), and our observations presented in this study suggest that diabetes plays a relatively less damaging role than smoking for CVD.

## 1. Introduction

Diabetes mellitus (DM) is defined as the group of metabolic diseases indicated by abnormal glucose levels in the blood over a long period of time. There are three types of DM: Type 1 DM is characterized by the loss of insulin-producing beta cells in the pancreas; it is traditionally termed a juvenile disease, as the majority of these cases are children and the disease is often inherited. Type 2 DM is indicated more by a poor responsiveness of the body tissues to insulin than by poor insulin secretion by the pancreas. This often occurs after middle age and is mostly caused by lifestyle choices, such as being overweight, eating an unbalanced diet, and having poor physical exercise habits. In addition, gestational diabetes occurs during pregnancy and is often resolved after childbirth. Out of these three, 90% of the 415 million DM patients fall under Type 2 [1,2]. Long-term, uncontrolled diabetes has been associated with Alzheimer’s disease and this is sometimes referred to as Type 3 DM [3].

DM leads to many serious complications, the most common being diabetic retinopathy, neuropathy, cardiovascular diseases, erectile dysfunctions, stroke, etc. According to the World Health Organization (WHO), a fasting glucose level of less than 6.1 mg/dL is normal, 6.1–7 is impaired glycemia or pre-diabetes, and above 7 is DM. More reliable and important ways of classifying different ‘shades’ of DM are based on HbA1C % (glycated hemoglobin %). It is a direct measurement of the glucose interaction with adult hemoglobin HbA. If this level is less than 6%, it is normal; if 6–7, it is pre-diabetes or a borderline case; if HbA1C > 7%, it is diabetes. Though it is well-known that DM is one of the major contributors to CVD, the damage done to erythrocytes per se has not received much attention, except for a few fragmentary reports [4]. The purpose of this report is to throw light upon the impact of excess glucose levels on erythrocytes in the case of pre-diabetic patients (a narrow range of borderline patients) and diabetic patients, plenty of which are found in the Kingdom of Saudi Arabia (KSA), a country with a median age of 25 years.

Two approaches were adopted: (1) atomic force microscopy (AFM). A few reports have appeared on the AFM images of the erythrocytes of diabetic patients, but a combined investigation has not appeared until now. This gives a much deeper insight into the impact of DM to look at the overall shape, size, and morphology (at the micrometer scale) and also surface roughness (at the nanometer scale) of erythrocytes; (2) spectroscopic analysis to measure the level of fluorescent biomolecules (tryptophan, NADH, porphyrin, etc.) in the blood plasma and cellular fractions on blood components which lead to CVD.

AFM is a scanning probe microscopy method by which the surface profile and morphology are measured by touching the surface with a sharp mechanical probe connected to a cantilever. The up and down movement of the cantilever as it walks through the ups and downs of the uneven surface, in a range of a few nanometers, is captured by a laser that is reflected off the head of the cantilever. In comparison to SEM, AFM can provide a 3D image of the surface profile [4,5,6].

Optical biopsy is an upcoming non-invasive procedure used to detect and diagnose certain diseases through the interaction of light/lasers with the biomolecules of tissue or body fluids. The ray of light that emerges from such interactions displays a certain change in wavelength due to Raman scattering and fluorescence. These two signals are fingerprints of biomolecules that are commonly found in tissue or fluid. The nature, quality, and quantity of biomolecules can be correlated to the specific disease of the subjects, as can be shown by the large number of studies on cancer tissues and the blood of inherited disorders [7,8,9,10,11,12]. The fluorescent spectral technique can be regarded as a new technique of molecular diagnosis. In an earlier report, similar work was done on smoking-induced hemolysis [13], and the present study can be considered an extension of such methods for monitoring the damage to erythrocytes due to diabetes mellitus.

## 2. Materials and Methods

In the present study, a total of 45 subjects participated: among them, 14 were pre-diabetic with HbA1C values of 6–7% (age range 20–38 and median age 30); the other 15 cases were diabetic with HbA1C values of 8–10% (median age 30). The remaining 16 were normal control subjects with a median age of 28. All of the control population of the present study were regular staff of the King Saud Medical City University Hospital, Saudi Arabia. The normal cases were selected based on exclusion criteria: the absence of interfering diseases. We explained the importance of the study to whoever participated. The work officially received approval from the institutional review board with the ethical committee no. E-12-754. All the subjects for this study were part of the non-smoking population.

The experimental protocol for this study was similar to that of our earlier reports [9,10,11,12,13]. A venous blood sample from each subject was collected in an Ethylene diamine tetra acetic acid (EDTA) tube. The collected sample was centrifuged at 3000 RPM for 15 min to separate the plasma and erythrocytes. The greenish-yellow supernatant plasma (1.5 mL) was drawn into a glass container and stored. Further, less than 1 mL of the buff coat was discarded with the help of a micropipette. A volume of 0.5 mL of a jelly-like thick component was extracted, washed with normal saline, and centrifuged at 3000 RPM for 10 min. This sample mostly contained erythrocytes and was taken for the AFM study. Next, another 0.5 mL of erythrocytes was collected and mixed with 1.5 mL of spectroscopic grade acetone. The sample was vigorously shaken and centrifuged at 3000 RPM for 15 min for the extraction of the fluorescent biomolecules in red blood cells (RBCs). The supernatant was taken for further spectral analysis.

An AFM (Multimode, Bruker, Santa Barbara, CA, USA) was used for this study. A small amount of the erythrocytes was poured and spread on sterilized round glass cover slips of a 12 mm diameter to make a monolayer. The sample was allowed to completely dry out before the AFM measurements. A silicon probe with an aluminum reflective coating on its back side (TEPSA, Bruker, Santa Barbara, CA, USA) was employed in AFM imaging. The probe has a spring constant of 20–80 N/m, a tip curvature with a radius of 8 nm, and a resonant frequency of 342–394 KHz. Furthermore, the AFM probe was used to visualize the top-view of the desired cells by an optical microscope. To analyze the alteration of the cell membrane, five to seven cells from different areas from each smear were randomly selected and scanned. The AFM images and roughness measurements were done by NanoScope Analysis 1.3 (Bruker, Santa Barbara, CA, USA).

The instrument for the spectral study was a spectrofluorometer (Perkin Elmer LS 55, Bruker, Santa Barbara, CA, USA). It is capable of measuring three different types of spectra, i.e., excitation, emission, and synchronous scans. The scanning range of the spectrophotometer was 200–800 nm. Blood plasma and erythrocytes have thousands of biomolecules, and of these, only specific fluorescent biomolecules, such as nicotinamide adenine dinucleotide (NADH), flavin adenine dinucleotide (FAD), porphyrin etc., were measured by this technique for this work. Based on the relative quantity of observed specific fluorescent biomolecules, the impact of DM on the blood was evaluated [7,8,9,10,11,12,13]. In order to see the individual impact of smoking or diabetes on RBC, the study was conducted with non- smoking diabetic patients and non-diabetic smokers separately.

## 3. Results

### 3.1. Morphological Analysis by AFM

**Figure 1:**
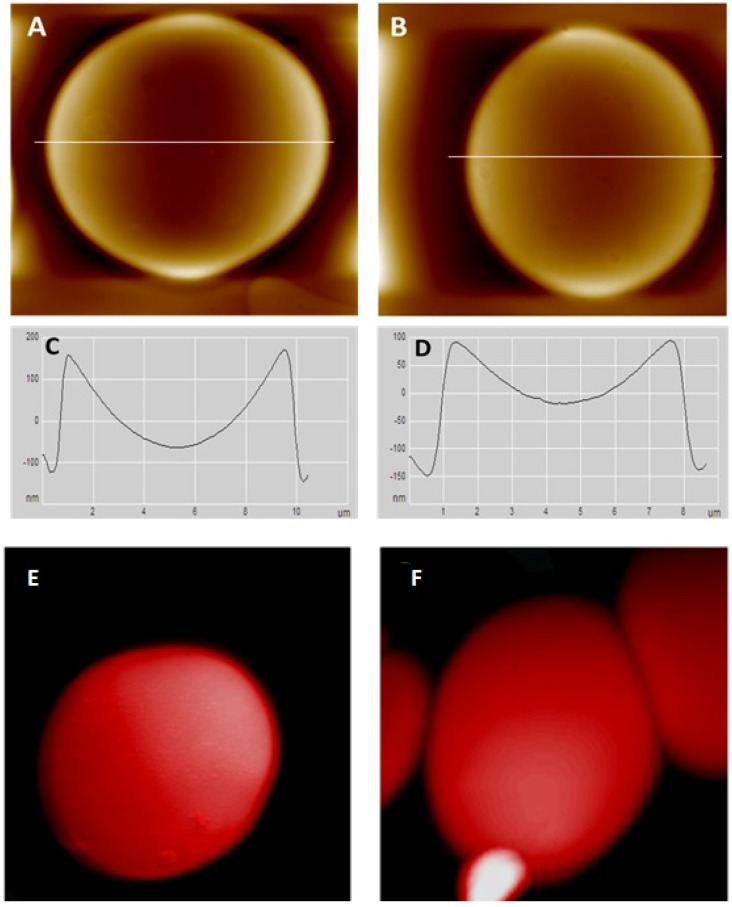
Images of erythrocytes of a normal (**A**) and pre-diabetic (**B**) individual by AFM (atomic force microscopy). (**C**) Geometrical profiles of the healthy control erythrocyte and (**D**) pre-diabetic erythrocyte. These cells are a representative of 20 smears. The diameter of the control cells is 8.13 ± 0.81 µm, and the diameter of diabetes cells is 6.87 ± 0.56 µm. The concave depth of pre-diabetic cells, 113 ± 46 nm, is only half of that of control cells, 267.1 ± 66 nm. Scan size = 10 × 10 µm. Also note the distinct, balloon-like structure of the RBC of the diabetic patient (**E**); the next important feature is the clinging and clustering of cells among themselves (**F**).

The figures illustrated here are representatives of a large number of observations made of a number of cells of each category. As shown in Figure 1A,B, the concave depth of a healthy normal RBC is 267.1 ± 66 nm, but it is only 113 ± 46 µm for an RBC of a pre-diabetic, as shown in Table 1. That is, the RBC becomes rather flat and also shrinks by 15% for pre-diabetics. Figure 1C,D indicate the concave curvature of cells from the above two categories.

This is to be compared to the shape of the RBCs of a diabetic subject, which visibly show a well-developed, inflated balloon-like, convex shape (Figure 1E). The size of the RBCs of these diabetic subjects is 9.11 ± 0.81 µm compared to the normal diameter of 8.13 ± 0.81 (*p* < 0.05), as shown in Table 1; i.e., almost 12% higher than the normal cells. The RBCs of diabetics have the tendency to stick together. Such changes must have been caused by the “glucose bath” that RBCs experience and by the glucose molecules that are able to seep through the membrane barrier and induce cytoskeletal changes. The overall result of such deformation and clustering retards the mobility of the blood flow [14].

**Figure 2:**
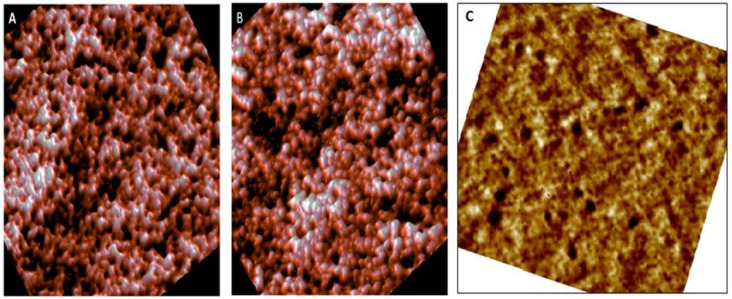
High-resolution images (Scan size is 900 × 900 nm) of the erythrocyte cell membrane taken by AFM for a healthy erythrocyte (**A**) and pre-diabetic erythrocyte (**B**). After filtering the images, the spectrin and actin network can be seen through the cell membrane. Mild pits and blowholes on the RBC (red blood cell) surface of diabetics (**C**). However, in the case of the diabetics, the surface is found with occasional blowholes and fissures, and the roughness is enhanced by about 15% (1.18 ± 0.60), as shown in Figure 2 and Table 1.

Figure 2 shows the surface of the erythrocyte membrane for control (A), pre-diabetic (B), and diabetic subjects (C). It can be seen that the surface roughness has not changed significantly for the pre-diabetic erythrocyte (1.05 ± 0.31 nm), compared to the control (1.07 ± 0.45 nm); see also data shown in Table 1.

The above results on the morphology of the RBCs for the pre-diabetic and diabetic subjects were compared with those of moderate and heavy smokers, published earlier by the same group [13]. All the smokers were non diabetic so that a comparison of the impact of smoking or diabetics on RBC could be made. Table 2 clearly shows that all the blood parameters are enhanced by 10 to 15 %, perhaps as a reaction to smoking.

**Figure 3:**
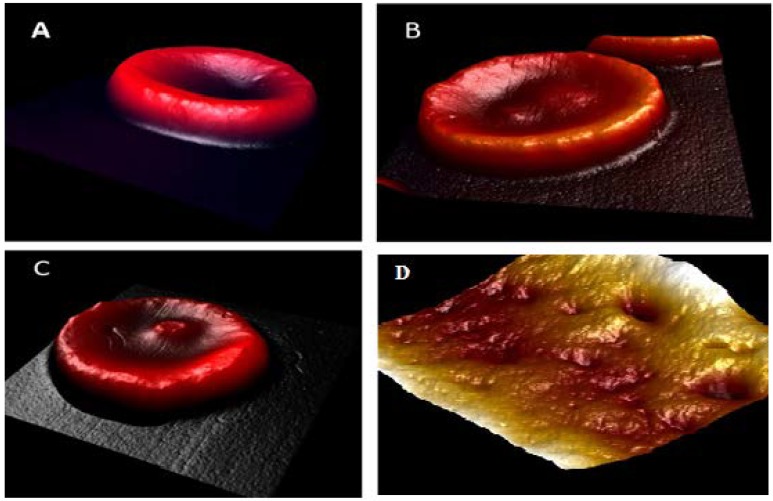
The RBC (red blood cell) shape by AFM (atomic force microscopy) investigation of the (**A**) normal control; (**B**) moderate smoker; (**C**) and heavy smoker; and (**D**) surface profile for a heavy smoker [13].

Figure 3A is the shape of the RBCs for the control, Figure 3B for the moderate smoker, and Figure 3C for the heavy smoker. Similarly, Figure 3D is the surface profile for a heavy smoker. A comparison among the AFM images of the RBCs of the normal controls, diabetic patients, and smokers reveals that, as far as the impact on RBCs is concerned, smoking is decisively more damaging than DM. See also Table 3, which shows drastic differences.

### 3.2. Spectroscopic Analysis

**Figure 4:**
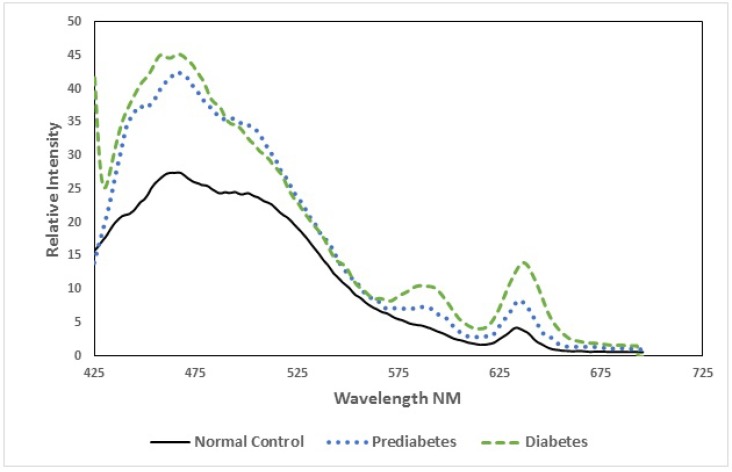
The fluorescence emission spectra (FES) of acetone extract of the red blood cells (RBCs) of (a) normal control, (b) pre-diabetic subjects, and (c) diabetic patients.

By convention, in fluorescence spectroscopy, the spectral intensities are presented in arbitrary units, as shown in Figure 4. There is a broad band at 475 nm (due to NADH), with secondary peaks at 585 nm and another at 635 nm (due to the basic and neutral species of porphyrin [8,9,10,11,12,13]).

**Figure 5:**
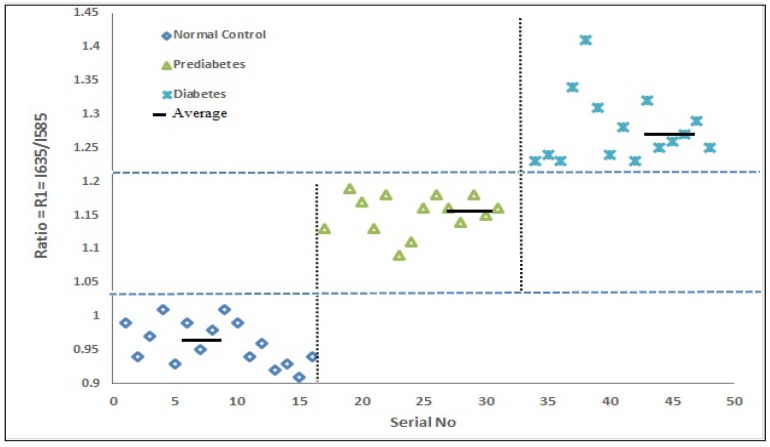
Scatter plot of R_1_ = I_635_/I_585_ values of the above three subjects; R1 is a measure of oxygen carrying capacity of red blood cells (RBCs).

Out of the three, the two peaks at 585 nm and 635 nm are important for our discussion. If we define the ratio R_1_ = I_635_/I_585_ as the intensity ratio for the two peaks, then it is 0.94 ± 0.01 for the normal subject, 1.15 ± 0.04 for the pre-diabetic subjects, and 1.25 ± 0.04 for the diabetic subjects. Also see the scatter plot of the R_1_ values shown for the above two sets (Figure 5).

It can be seen that there is about a 15% increase in the porphyrin content for pre-diabetics and a 25% increase in diabetic subjects compared to the control. This could be due to the higher content of hematocrit often associated with diabetics [15]. The synchronous emission spectra (SES) of plasma give a measure of essential amino acids like tryptophan, NADH, FAD, and other biomolecules present in the plasma. These fluorescent biomolecules are essential for the proper functioning of RBCs and other cells of the tissue. When the delicate balance necessary for wellness is upset, these molecules become out of proportion, as shown below.

**Figure 6:**
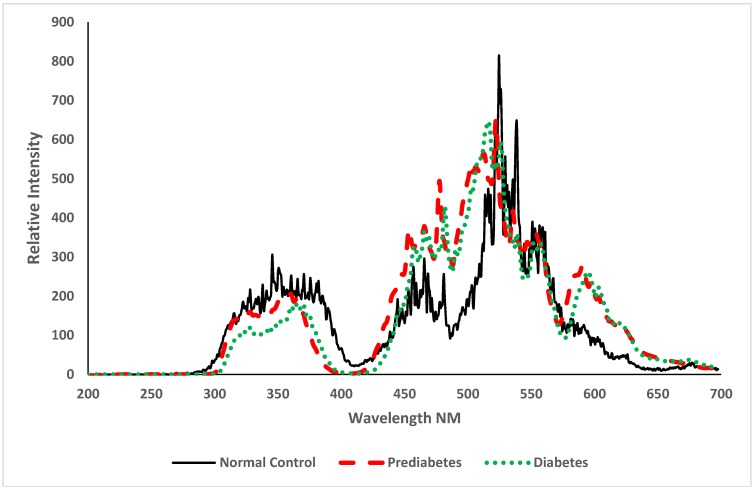
Synchronous emission spectra (SES) of blood plasma of (a) normal control, (b) pre-diabetic subjects, and (c) diabetic patients. SES was obtained by keeping an offset of 10 nm between the excitation and emission grating.

Figure 6 is the SES of the plasma of controls compared to those of pre-diabetic and diabetic subjects. Here, the peak at 360 nm is due to the essential and ubiquitous amino acid tryptophan, the peak at 460 nm is due to NADH, and the peak at 525 nm is for FAD.

**Figure 7:**
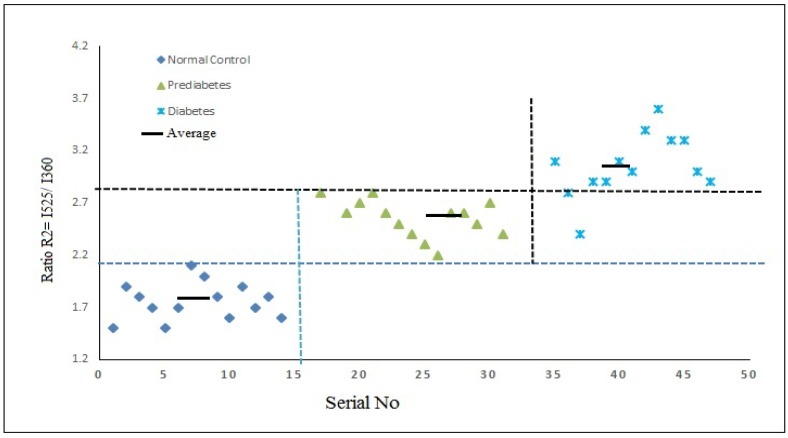
Scatter plot of R_2_ = I_525_/I_360_ values of the above three subjects. R_2_ is a measure of the redox reaction and decay rate of any cell—in this case, RBCs (red blood cells).

The ratio parameter R_2_ = I_525_/I_360_ is almost 1.77 for normal, 2.6 for diabetic, and 3 for diabetic subjects (Figure 7). The ratio is an indication of excessive oxidative stress, as indicated by the twice-enhanced concentration of FAD. This is expected, as the coenzyme FAD is involved in the redox activity of many cellular processes, and tryptophan is involved as a building block material in healthy cells. Thus, the ratio R_2_ is a measure of the decay of any tissue—in this case, RBCs. As can be seen from the figures, uncontrolled diabetes leads to a faster decay of tissues due to excessive oxidative stress.

Tryptophan is an essential amino acid involved in every cellular activity. The very fact that the level of this amino acid is about 10–30 lower for pre-diabetics and diabetics is a strong indication that the tissues have poor nutrients. When the glucose level exceeds the optimum value, leading to pre-diabetes and then to diabetes, cells get fewer and fewer nutrients and end up exhibiting starvation and eventual dysfunction.

## 4. Discussion

The most important contributory factors for cardiovascular disease are smoking, diabetes mellitus, hypertension, and plaque in arteries. Type 2 diabetes mellitus (DM) is an independent risk factor for developing CVD, with at least a 1.7 times greater risk relative to non-diabetic subjects [15,16,17,18,19,20,21]. Also, DM is the leading cause of erectile dysfunction, which may be regarded as a clinical marker for CVD. The main objective of the present study is to give a fairly close view of the relative damage done by the first two, based on spectroscopic and microscopic analyses.

It is important to draw attention to a number of studies done on the impact of smoking on the RBCs of non-diabetic and diabetic subjects. In a detailed study by Agarry et al. it was shown that the nicotine and cotinine of smoke produce oxidative stress, and breaking of the –SH bond of the RBC membrane leads to a 20% higher rate of hemolysis [22]. Also, the study showed that smoking leads to persistent hypoxia, due to which the erythrocyte count is enhanced by 10% [23]. Smokers have not only an enhanced RBC count: WBC, lymphocyte, and monocytes counts are also enhanced by 6–10% [24]. The nicotine of smoke enhances clots in coronary arteries and leads to endothelial dysfunction. The free radicals and peroxides of smoke are linked to the pathogenesis of atherosclerosis and carcinoma. Another study has reported that smoking diabetic patients have a 12% greater HbA1c level (9%) than the controls; for non-smoking diabetics, this figure is 8% [25].

DM leads to a long chain of complex metabolic changes, and the actual mechanism establishing a proper correlation between DM and CVD is not known yet [26,27,28,29]. Numerous studies have revealed the mechanisms underlying the vascular dysfunction that leads to cardiovascular outcomes in DM. The disease of the blood vessel is related to the drastic reduction of nitric oxide (NO) and prostacyclin [30,31,32]. To this complex scenario, the investigations described in this paper give an improved insight.

When glucose is left unutilized in the blood circulation, RBCs seem to be immersed in a “glucose bath”, converting the biconcave “ring-like” structure of RBCs into a flat ring and then into a convex balloon. Glucose is a small molecule and can easily seep through the erythrocyte membrane and bloat from inside. Such stage-by-stage disease progression can be seen by AFM. Since the average size of an RBC is 10–15% bigger in diameter, the individual RBC volume becomes 53% more for diabetes subjects. (As the radius of the diabetic RBC increases by 15%, the volume of each RBC increases by 53% (1.15 × 1.15 × 1.15) in comparison to the normal control).

This may be the main cause for the increased viscosity of blood in diabetic patients. In addition, these RBCs appear to have a tendency to stick together, and this could be the reason for the enhanced coagulation attributed to DM. Because of the reduced mobility of RBCs and their clumping together, they end up with higher friction among themselves and also when passing through micro-vessels. This has two deleterious effects: one damaging effect is to the RBC itself, which is supported by the spectroscopic investigation, particularly the ratio R_2_, which is a measure of a high concentration of FAD, a coenzyme of decay products of any cell—in this case, RBCs. The debris of RBCs thus obtained results in sedimentation and promotes plaques in cardiovascular arteries, leading to CVD and distal organ dysfunction, causing erectile dysfunctions and retinopathy [33,34,35,36].

These kinds of damages are true for smokers too [13,22,37]. However, there are certain important differences, as shown by the AFM images in this paper and in our earlier work. The RBCs of a smoker become pitted by the toxic gases and particulate matter of smoke. This leads to greater surface roughness and friction among themselves and with the walls, leading to both debris and sedimentation on the walls of micro-vessels. The noteworthy point here is that the glucose-induced damages to the morphology of RBCs by DM appear to be significantly less than those caused by smoking. In addition, smoking leads to a heavy reduction in R_1_, which is a measure of the oxygen carrier porphyrin [13]. This value is 40% lower for smokers, but 20% higher for DM patients, than for normal controls. That is, DM patients will not suffer from a lack of oxygen being delivered to different tissues, but they may suffer more from high viscosity. This may lead to additional adverse effects, such as increased platelet expression and thrombosis. In spite of all these problems, the present study indicates that a medium smoker (arbitrarily defined as five cigarettes per day for a three-year period) is at a much higher risk of cardiovascular trauma than a medium glucose-level diabetes patient (a subject who controls their blood sugar level at 7 mM/L for a period of three years by diet, medicine, and exercise). This could be one of the reasons for less CV trauma in the female than male population. DM is significantly more common among women after middle age, but most of them do not smoke. In contrast, smoking is quite common among middle-aged men, particularly non-sedentary workers like drivers or carpenters. These men generally are not diabetic and are physically active, yet end up with unexpected cardiovascular trauma, all because their R_1_ is much lower and R_2_ is much higher than in subjects with DM. However, when the subject is a smoker and diabetic, the risk for CVD is much higher due to any one of them alone, as they seem to have synergism in causing damages.

## 5. Conclusions

In this preliminary study of atomic force microscopic and spectral analyses on the impact of diabetes on erythrocytes, it is shown that excess glucose in the blood leads to a significant deformation of the shape of RBCs and mild corrosion on their surface because glucose can pass through the membrane and surround it like an envelope. There are two abnormal results. One is that RBCs become bloated, and the other is that the “glucose bath” helps the cells to stick together, as sugar is an adhesive. Both eventually lead to enhanced viscosity and retarded mobility for RBCs. Further, as glucose is corrosive, the erythrocyte becomes weaker and weaker from the outside and inside. A third noteworthy result is the suppression of essential amino acids in plasma, leading to the deprivation of proper nutrients to tissues and cells. The study was designed to provide insights into the impact on RBC exclusively by diabetics or smoking, and it shows that RBC appears to be less damaged by diabetes than by smoking. In other words, a non–diabetic, long-term smoker is more likely to suffer from cardiovascular diseases than a non–smoking, long-term diabetic patient.

## Figures and Tables

**Table 1:** Statistical analysis of some erythrocyte parameters of the pre-diabetic, diabetic, and healthy individuals with significance *p* < 0.05.

Parameter	Normal (Control)	Pre-Diabetic	Diabetic
Diameter (µm)	8.13 ± 0.81	6.87 ± 0.56	9.11 ± 0.81
Concave depth (nm)	267.1 ± 66	113 ± 46	convex
Roughness (nm)	1.07 ± 0.45	1.05 ± 0.31	1.18 ± 0.60

**Table 2:** Statistical analyses of demographic and laboratory value of data of normal control, smokers (male), and smokers (female).

Hematological Parameters	Normal Control (*n* = 31)	Smokers (Male) (*n* = 21)	Smokers (Female) (*n* = 10)
Hemoglobin (g/L)	165 ± 0.95 (normal range male 130–180) 139 ± 1.15 (normal range female 120–160)	171.13 ± 1.09	143.18 ± 1.38
Hematocrit (%)	41 ± 0.59 (normal range male 42–52); (normal range female 37–47)	43.25 ± 2.01	39.47 ± 0.98
Red blood cell (RBC, ×10^12^/L)	4.9 ± 0.45 (normal range male 4.7–6.1) 4.4 ± 0.31 (normal range female 4.2–5.5)	5.02 ± 1.12	4.71 ± 1.18
Mean corpuscular volume (MCV) (fL)	85 ± 1.12 (normal range 80–94)	89.12 ± 1.52	86.4 ± 1.45

**Table 3:** Statistical analysis of parameter of the roughness values of the RBCs (red blood cells) of normal, moderate, and heavy smokers, and pre-diabetic and diabetic subjects.

Parameter	Normal (Control)	Moderate Smokers	Heavy Smokers	Pre-Diabetic	Diabetic
Diameter (µm)	8.13 ± 0.81	10.3 ± 1.06	Irregular shape	6.87 ± 0.56	9.11 ± 0.81
Concave depth (nm)	267.1 ± 66	296 ± 33	80 ± 41	113 ± 46	convex
Roughness (nm)	1.07 ± 0.45	1.30 ± 0.3	5.5 ± 3.0	1.05 ± 0.31	1.18 ± 0.60
